# Health System Resilience and Afghan Refugee Health in Pakistan: An Exploratory Study of Challenges and Pathways to Inclusion

**DOI:** 10.1002/hpm.70113

**Published:** 2026-07-28

**Authors:** Zahid Ali Memon, Asma Ummad, Amina Olabi, Natasha Palmer, Sophie Witter

**Affiliations:** ^1^ Department of Community Health Sciences Aga Khan University Karachi Pakistan; ^2^ Institute for Global Health and Development Queen Margaret University Edinburgh UK

**Keywords:** afghan refugees, health system resilience, Pakistan, primary healthcare, refugee health policy, service delivery, universal health coverage

## Abstract

Pakistan has hosted Afghan refugees for more than four decades, in one of the world's most protracted displacements of people. Afghan refugees receive health services through United Nations High Commissioner for Refugees (UNHCR) and non‐governmental organisation (NGO) supported programmes alongside public sector facilities at every tier. They remain excluded from key national health protection programmes such as the Sehat Sahulat Programme. Understanding the system‐level enablers and barriers is essential for advancing equitable and sustainable healthcare for refugees and host populations. An exploratory qualitative case study was conducted using 30 key informant interviews and three participatory Group Model Building (GMB) workshops across Khyber Pakhtunkhwa, Sindh, and Islamabad Capital Territory. Data were thematically analysed using a Health System Resilience and Refugee Response Framework. Findings reveal a mixed picture of resilience and fragility. Refugees with Proof of Registration and Afghan Citizen Cards reported using primary care on a par with residents, while high reported immunisation coverage was widely perceived to have reduced polio risk among Afghan refugee children since around 2014, in line with national data showing a sharp decline in wild poliovirus cases over this period. While essential health services are being provided to refugees in the camps, they continue to face critical shortages, including frequent stock‐outs of medicines, limited diagnostic capacity, too few trained female healthcare providers, and fragmented referral pathways. Financing remains heavily donor‐dependent, with refugees excluded from the Sehat Sahulat Programme, the National Health Insurance programme. Governance challenges, including overlapping mandates, poor coordination, and a lack of refugee‐specific data, undermine efficiency and planning. Despite these challenges, community health workers and shared health initiatives fostered social cohesion and equitable treatment in public sector health facilities. Our study demonstrates that inclusive health services can be delivered in Pakistan at scale but cannot be sustained and scaled without broader health system strengthening reforms. The inclusive and resilient refugee health services require embedding refugee health into national strategies, ensuring predictable financing, stronger coordination mechanisms, and integrating refugee data into health management information systems. These findings offer lessons for other host countries managing protracted refugee crises that create substantial challenges for national health‐system financing, workforce, and service delivery.

## Highlights

- Pakistan has hosted Afghan refugees for over four decades and many of them without legal status.
- Immunisation expanded coverage, cutting polio incidence from its 2014 peak.
- Donor‐dependent financing leaves refugees outside Sehat Sahulat coverage.
- Inclusion in national health systems is vital for equity in protracted crises.

## 1 Introduction

The worldwide refugee crisis has reached an unprecedented scale, with over 120 million people now displaced and a disproportionate burden falling on the health systems of host countries [1]. Refugee health faces numerous challenges, including exclusion from national health systems, limited funding, and reliance on donor‐driven services, particularly in low‐ and middle‐income countries [2]. Pakistan has hosted large numbers of Afghan refugees displaced since 1979 by conflict and political instability [3]. Afghan displacement into Pakistan occurred in three distinct waves. The first began with the Soviet invasion in 1979, with over 400,000 Afghans arriving initially; by the mid‐1980s, the combined refugee population in Pakistan and Iran exceeded five million [4]. A second wave followed the post‐9/11 period; a 2005 national census counted approximately three million Afghans in Pakistan, leading to the issuance of over 1.5 million Proof of Registration (PoR) cards by 2007 [5]. The most recent influx was triggered by the Taliban takeover on August 15, 2021, with an estimated 600,000–800,000 additional Afghans arriving by 2023 [6]. In September 2023, Pakistan issued the Illegal Foreigners Repatriation Plan, resulting in over 200,000 Afghans departing in November 2023 alone [7]. Most Afghans in Pakistan reside in Khyber Pakhtunkhwa (KP) and Balochistan, with smaller but significant populations in Punjab and Sindh [8]. Yet after nearly five decades of continuous Afghan presence, the legal and institutional framework governing their stay has never settled into a stable architecture, and this instability shapes every layer of their access to healthcare.

Pakistan is not a signatory to the 1951 Refugee Convention or its 1967 Protocol and lacks national refugee protection laws [9]. This legal vacuum leaves Afghan refugees without formal rights, increasing their vulnerability to deportation, discrimination, and restricted access to essential services such as healthcare, education, and livelihoods [10]. The Commissionerate for Afghan Refugees (CAR) works with provincial governments, the United Nations High Commissioner for Refugees (UNHCR), and NGOs to facilitate service delivery, particularly in refugee villages; however, these arrangements remain fragmented and insufficient to ensure long‐term inclusion [11]. Although international partners such as UNHCR contribute to refugee support, host‐country health and social systems shoulder the bulk of the cost and administrative burden in Pakistan [12, 13]. UNHCR's role has shifted from providing camp‐based healthcare services to facilitating refugee access to public facilities, yet evidence from low‐ and middle‐income countries indicates that substantial gaps in coverage and coordination often persist [14].

Beyond this institutional picture, the lived position of Afghan refugees in Pakistan has also shifted. Over time, many refugees have transitioned from camp‐based living to urban and peri‐urban settlements, contributing to the informal economy [15]. Yet their socio‐economic integration remains limited due to restricted legal status [16]. Global evidence highlights that migrants and refugees face multi‐layered obstacles to achieving universal health coverage, including legal exclusion, documentation requirements, financial barriers, administrative delays, and language and cultural barriers, with undocumented and irregularly staying populations facing the greatest burden [17].

Taken together, these institutional arrangements and lived realities highlight how protracted refugee situations strain national health systems and underscore the need for sustainable models that integrate refugee health within broader public health structures [18]. Despite repeated international calls for such integration, refugee health systems often remain donor‐dependent, fragmented, and weakly integrated into national frameworks [19]. Ensuring sustainability requires transitioning towards nationally led, inclusive models that align with universal health coverage goals. Against this backdrop, Pakistan, hosting one of the world's largest and protracted refugee populations, offers a critical case for examining how national systems adapt to meet refugee health needs under resource constraints. The question that animates this study follows directly from the legal, financial, and institutional context laid out above: how does Pakistan's health system provide healthcare access to Afghan refugees despite the absence of formal refugee legislation, exclusion from national social health protection schemes, and reliance on donor‐dependent financing mechanisms?

This study applies a health systems framework to examine how service delivery, governance, financing, and information systems interact to shape inclusiveness, equity, and resilience in refugee healthcare across provinces. Conducted as part of a UNHCR‐commissioned multi‐country research programme on health system resilience and refugee responses, this case study aims to generate actionable insights for policymakers and donors to strengthen coordination, integration, and long‐term sustainability of refugee health services in Pakistan, while contributing to the broader global movement towards refugee health system integration.

## 2 Methods

### 2.1 Study Design

This study employed an exploratory qualitative case study design guided by the Health System Resilience and Refugee Response Framework (Figure 1), developed by Queen Margaret University for cross‐country analysis of six country case studies [20]. The framework depicts the health response as a multi‐stage process moving left to right across four interconnected domains: context, key barriers, health system functions, and outputs and outcomes.

**FIGURE 1:**
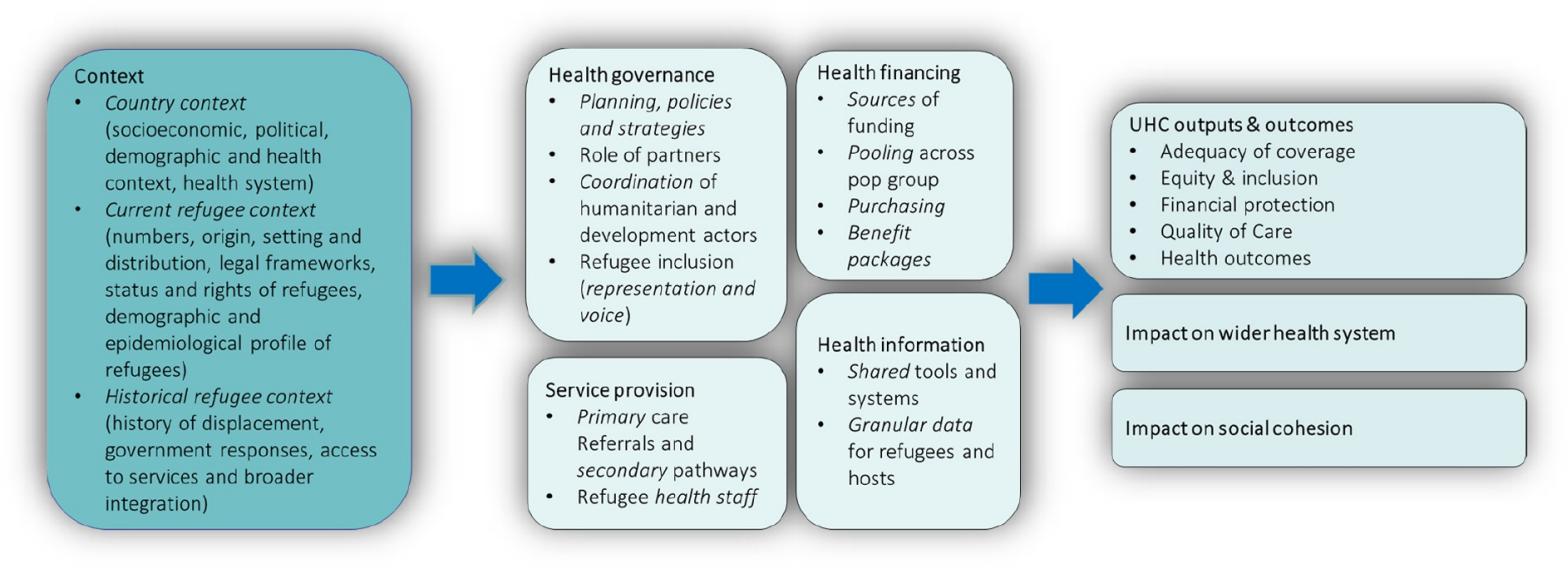
Health system resilience and refugee response framework.

The framework arranges the refugee health response as a left‐to‐right system pathway across three zones. The contextual zone (left) covers the refugee context (legal status, displacement history, numbers, distribution), the country context (legal provisions, fiscal space, political settlements), the local context (socio‐economic conditions, livelihoods, settlement patterns), and the health context (epidemiological profile, unmet needs, social determinants). The system functions zone (centre) covers governance, financing, service provision (including human resources for health), and monitoring, evaluation, and learning. The outputs and outcomes zone (right) covers Universal Health Coverage (UHC) outputs and outcomes for refugees and host populations: adequacy of coverage, equity and inclusion, financial protection, quality of care, and health outcomes, together with wider system effects (facility strain, sustainability) and social cohesion. The framework guided the development of the topic guides, the codebook, and the integration of findings.

### 2.2 Site Selection

In Pakistan, refugee hosting falls under the federal jurisdiction, whereas healthcare is the responsibility of provincial governments. Afghan refugees are primarily concentrated in KP, Balochistan, Sindh, and the Islamabad Capital Territory (ICT), which differ in demographic composition, service delivery arrangements, and access to care, shaping how refugee populations are distributed and which services they can reach. Study sites were selected in collaboration with UNHCR Pakistan, whose extensive operational presence and data on refugee populations ensured the inclusion of settings representing diverse socio‐economic contexts, health system structures, and patterns of healthcare utilization, while also introducing potential selection bias towards areas with stronger UNHCR presence. Partnering with UNHCR enabled ethical and authorised access to refugee communities and facilitated coordination with the CAR. Their involvement also supported the study's site selection process, ensuring that selected locations were operationally accessible and methodologically relevant, a consideration we reflect on as a limitation when interpreting the findings. The study sites included Peshawar and Kohat in KP, which host large and long‐established Afghan refugee populations situated near urban health facilities; Karachi in Sindh, representing a densely populated urban setting with diverse livelihood opportunities but persistent gaps in healthcare access; and the Islamabad Capital Territory, notable for its proximity to national policy institutions and international organisations, as well as its mixed refugee population.

### 2.3 Data Collection

#### 2.3.1 Document Review

The desk review examined existing evidence on healthcare for Afghan refugees in Pakistan, drawing on peer‐reviewed journal articles, national and provincial policies and plans, UNHCR and partner reports, NGO publications, and other grey literature. A structured search of PubMed and Google Scholar (2017–2024) used combinations of terms such as “Pakistan*” AND (‘health’ OR ‘health system’ OR ‘governance’ OR ‘health financing’ OR ‘health service’) AND (‘refuge*’ OR ‘migrant*’ OR ‘displace*’) AND (‘social cohesion’ OR ‘integration’ OR ‘trust’), complemented by hand‐searching UNHCR and NGO websites and news sources for additional reports and datasets. After screening 286 database records and grey sources, 82 documents met predefined inclusion criteria, focus on Afghan refugees or mixed refugee‐host populations in Pakistan; information on health policies, service delivery, financing, governance, data systems, social cohesion, or health outcomes; English language, and underwent thematic analysis to map institutional arrangements, financing flows, service delivery mechanisms, and key health status patterns, which in turn informed sampling and topic guides for the primary qualitative work.

#### 2.3.2 Key Informant Interviews (KIIs)

Thirty semi‐structured KIIs were conducted between February and March 2024 with stakeholders at national, provincial, and community levels. Participants included officials from health departments, UN agencies, NGOs, Afghan community representatives, and healthcare providers. Interview questions were structured around the Health System Resilience and Refugee Response Framework, focussing on contextual factors and core health system functions, including service provision, financing, governance, and monitoring and evaluation. All interviews were conducted face‐to‐face after obtaining written or verbal informed consent from participants, and detailed notes were taken to capture contextual nuances. The 30 interviews were distributed across the three study sites: 15 in Khyber Pakhtunkhwa (KP), 6 in Sindh, and 9 in ICT, purposively selected to represent diverse stakeholder categories including government health officials, UN agencies, INGOs, NGOs, Afghan Commissionerate representatives, healthcare providers, and Afghan refugee community members, with a larger number in KP to reflect its higher concentration of Afghan refugees and wider range of implementing partners. The profile of KII participants by stakeholder category and site is presented in Table 1.

**TABLE 1:** Profile of KII participants by stakeholder category and site.

Stakeholder category	KP	Sindh	ICT	Total
Provincial department of health	4	1	1	6
Commissionerate for afghan refugees (CAR)	2	0	1	3
United nations agencies (UNHCR, UNFPA, UNODC)	2	1	3	6
International and national NGOs (IRC, IOM, IDEA, SRSP, GIZ, RAHA)	4	2	3	9
Healthcare providers (MALC, green star)	0	1	1	2
Afghan refugee community members	2	1	1	4
Total	14	6	9	30

Abbreviations: GIZ, Deutsche Gesellschaft für Internationale Zusammenarbeit; IDEA, Initiative for Development and Empowerment Axis; IOM, International Organisation for Migration; IRC, International Rescue Committee; MALC, Marie Adelaide Leprosy Centre; RAHA, Refugee Affected and Hosting Areas programme; SRSP, Sarhad Rural Support Programme; UNFPA, United Nations Population Fund; UNODC, United Nations Office on Drugs and Crime.

#### 2.3.3 Group Model Building (GMB) Workshops

Three participatory GMB workshops were conducted between May and June 2024, one each in KP, Sindh, and ICT, to complement interview findings. Participants included refugees, host community members, service providers, and local health managers. Using systems‐thinking exercises such as rich pictures, variable elicitation, and causal loop diagraming [21], participants collaboratively mapped interlinkages between contextual and system factors influencing refugee healthcare. Each workshop was structured to aim for diverse gender and stakeholder representation, with participants drawn from refugee community members, health facility staff, government departments, and the Commissionerate for Afghan Refugees, United Nations agencies, and international and national NGOs. The composition of GMB workshop participants by site is presented in Table 2.

**TABLE 2:** Composition of GMB workshop participants by site (*n* = 38).

Site	Provincial DoH	CAR	UN agencies	INGOs/NGOs	Healthcare providers	Refugee community	Total
KP (peshawar and kohat)	1	2	2	6	1	4	16
Sindh (karachi)	1	0	1	3	1	2	8
ICT (islamabad)	1	1	3	8	0	1	14
Total	3	3	6	17	2	7	38

### 2.4 Data Management and Analysis

Interviews were transcribed verbatim and translated into English. Field notes from GMB sessions were compiled and triangulated with KII transcripts. Documents identified in the desk review, including policies, programme reports, and published studies, were imported into the same analytic framework and coded deductively against the Health System Resilience and Refugee Response domains, with inductive codes added for emergent themes. Where available, routine secondary data on health service utilization and key indicators for refugee‐hosting areas were extracted and summarised descriptively to contextualise qualitative findings and to assess data gaps. An interpretive thematic analysis was conducted, combining deductive coding guided by the Health System Resilience and Refugee Response Framework and inductive coding emerging from the data. Three members of the research team were involved in the coding process. Each independently coded a subset of transcripts, followed by team discussions to reconcile differences and achieve consensus on code definitions and theme interpretation. Data from KIIs and GMB were integrated during analysis to identify converging and diverging perspectives. Causal loop diagrams developed during GMB sessions were validated against interview findings, enabling a systems‐level understanding of feedback mechanisms within the refugee health system.

### 2.5 Ethical Considerations

Ethics approval for primary data collection for all case studies was obtained from Queen Margaret University (UK), followed by approval from the Aga Khan University Ethics Review Committee (AKU‐ERC; ID: 2023‐9185‐26641), followed by the National Bioethics Committee (NBC), Pakistan. Data were anonymised during transcription, and identifiers were removed before analysis. All interviews and workshops were conducted in private spaces, with assurances that individual responses would be kept confidential and reported only in aggregated or anonymised form. Recordings and transcripts were stored on encrypted, password‐protected devices accessible only to the research team.

## 3 Results

The findings are organised around the analytical framework, beginning with contextual factors, followed by health system functions, and concluding with UHC outputs and outcomes. Evidence from documents, KIIs, and GMB workshops is integrated thematically rather than separated by data source. Document review provides the principal evidence for contextual findings, while KII and GMB workshops carry greater weight in the health system functions and outcomes sections.

### 3.1 Context: Refugee History, Legal Status, and the Pakistani Health System

Pakistan currently hosts approximately 3.1 million Afghans, including around 1.35 million Proof of Registration (PoR) card holders and more than 800,000 Afghan Citizen Card (ACC) holders, while a large residual population remains unregistered [22]. Pakistan is not a signatory to the 1951 Refugee Convention or its 1967 Protocol and has not enacted national refugee protection legislation; refugee protection therefore rests on administrative arrangements and bilateral cooperation rather than on an enforceable rights framework [9]. The PoR card, introduced in 2006 and issued jointly by the Government of Pakistan and UNHCR to registered Afghan refugees, provides temporary legal stay, freedom of movement, and relatively secure access to public services such as health and education. In contrast, the Afghan Citizen Card (ACC), introduced in 2017 and issued by NADRA to previously undocumented Afghan nationals, grants temporary documentation and proof of identity but offers weaker protection and less reliable access to services than the PoR, leaving holders at greater risk of repatriation [23] PoR holders may use public health facilities and the Expanded Programme on Immunisation (EPI), tuberculosis (TB), and HIV services on the same terms as Pakistani citizens; ACC holders access these services in practice but without legal guarantee. Both groups are excluded from Computerised National Identity Card (CNIC) linked social protection schemes, including the Sehat Sahulat Programme (SSP) and the Benazir Income Support Programme (BISP), because eligibility is tied to citizenship‐based national identity documentation [24].

Pakistan's public health system is organised as a decentralised three‐tier structure comprising tertiary teaching hospitals, secondary District Headquarters and Tehsil Headquarters hospitals, and a primary care network of Basic Health Units (BHUs), Rural Health Centres (RHCs), dispensaries, and community‐based Lady Health Workers [25]. The 18th Amendment in 2010 transferred most health functions to provincial governments but did not equalise fiscal capacity across provinces [26]. Total health expenditure has hovered around 2.9% of GDP, among the lowest in the region, and public per capita expenditure falls below WHO‐recommended thresholds [27]. District and tehsil hospitals routinely face medicine shortages, staffing deficits, and equipment dysfunction regardless of refugee presence [28]. The 2022 UNHCR Health Access and Utilisation Survey (HAUS) found that more than 90% of refugees pay out of pocket for healthcare and nearly 70% are unaware of subsidised public services, sustaining reliance on private providers [29]. UNHCR facility‐mapping data from KP indicate that 78% of Refugee Villages lie within 8 km of a public health facility and 22% within 9–12 km; nominal geographic proximity does not remove transport, affordability, or mobility barriers, particularly for women and daily‐wage earners [30]. Refugees are therefore layered onto an already constrained public system, and the system effects observed in this study are best read as the interaction between an under‐resourced public sector and the additional legal, documentation, and financial barriers refugees face.

Interviews and GMB workshops triangulate the documentary record on policy trajectory. Participants across the three sites described four broad phases. A humanitarian‐emergency phase from 1979 to 1989 was financed largely by UNHCR through parallel BHU‐based services, with free medicines, ambulances, and door‐to‐door preventive care. A transition phase from 1989 to 2009 saw service‐led financing peak between 1994 and 2009, supported by strong UNHCR and WHO coordination and tailored TB, maternal, neonatal, and child health (MNCH), and nutrition programmes. A contraction phase from 2009 to 2014 followed, in which donor fatigue and the diversion of funds to Syrian, Yemeni, and other emerging crises produced the closure of camp‐based labour rooms in 2009 and the staged withdrawal of UNHCR financing for medicines. An integration phase from 2014 to 2024 opened with the deliberate redirection of foreign funding from dedicated refugee facilities to general public sector strengthening through the RAHA programme [31]. While intended to support integration, KP and ICT GMB participants argued that the shift left designated refugee facilities under‐resourced and contributed to the collapse of door‐to‐door services by 2014.

### 3.2 Governance and Coordination

Refugee health governance in Pakistan is multi‐actor but fragmented. Provincial health departments hold formal responsibility for policy direction and service oversight; the CAR, UNHCR, World Health Organisation (WHO), and other international agencies provide financing, technical input, and programmatic oversight; and national and international NGOs operate as the principal implementers of clinics, mobile health units, and community‐based programmes. Across the three sites, participants converged on the view that this configuration is project‐driven rather than institutionally embedded and that short funding cycles undermine continuity:

NGOs come with good services, but their projects are short‐term. When one project ends, another starts with similar objectives, sometimes overlapping in the same areas. This creates confusion and breaks continuity in services.

(KP KII‐06)

Coordination is described as inefficient. Inter‐agency working groups exist on paper, but parallel meetings, inconsistent communication, and bureaucratic delays were reported across all three sites. The CAR and the provincial departments of health typically convene separate meetings rather than a single platform:

Currently, the Afghan Commissionerate conducts its own meetings while the health department carries out separate meetings, which is not conducive to effective collaboration. It’s imperative that they come together at the same table to ensure coordination.

(KP KII‐02)

A community‐level GMB account captured the same fragmentation in plainer terms:

Earlier, different organisations worked together. Now everyone is working in silos, and no one knows who is responsible for what.

(KP‐GMB)

The reported consequences are operational: misallocated insecticide stocks, short‐expiry paracetamol supplies, slow approval cycles for No Objection Certificates from the Ministry of States and Frontier Regions, and equipment that lies unused for want of staff or for want of interest. A KP‐based key informant described both the coordination gap and its tangible logistical outcomes:

The Afghan Commissionerate has gaps in coordination among various stakeholders. For example, when we requested insecticide and bed nets, they were not provided in the required number. Moreover, we received paracetamol with a short expiry date.

(KP KII‐02)

The unpredictability of permissions adds a further layer of friction, with one informant noting that approvals depend less on procedure than on personal disposition:

Obtaining permissions and NOCs from other entities, such as the Ministry of Saffron, the Chief Commissioner, and the Afghan Commissionerate, is a more cumbersome process. It often involves significant delays, which are influenced by the mood of government officials.

(KP KII‐11)

Accountability weaknesses compound this fragmentation. Resource use is rarely tracked even when equipment is in place, and informal payments and administrative delays were reported at multiple steps of approval:

There is little follow‐up on how resources are used. Even when equipment is available, it often lies unused due to a lack of interest or staff. Administrative delays is another big problem; undue favours are expected at every step, even for signing an MoU.

(KP KII‐08)

Political turnover repeatedly resets ongoing initiatives. The KP GMB workshop pointed to the absence of a fully active central coordinating body and to cumulative absorption of funds at successive administrative tiers as recurrent causes of dispersal; the Sindh and ICT workshops described the same pattern, though the CAR is more visible in KP than in the other two sites.

### 3.3 Health Financing

The principal sources of finance for refugee health are donor funds, UNHCR and other UN agencies, bilateral donors including the World Bank, United States Agency for International Development (USAID), and WHO, and private donors, channelled through implementing partners; provincial public sector budgets into which refugee‐serving facilities are folded without a dedicated line; and emergency cash assistance from UNHCR through the CAR, capped at PKR 45,000 per family for acute medical situations. Pakistan has no dedicated government budget line for refugee healthcare. GMB workshops in KP, Sindh, and ICT converged on a single message: operations have been funds‐driven, with no sustainability factor built in. UNHCR funds for implementing partners are restricted to infrastructure, equipment, and capacity‐building, and cannot be used for recurrent expenditures such as medicines, vaccines, or staff salaries; as donor funding contracted, BHUs were progressively staffed with less‐qualified personnel. A Sindh GMB participant articulated the policy void that drives this volatility:

Funding is always uncertain, and without proper policies, services for refugees are inconsistent.

(Sindh‐GMB)

Workshop trajectories, KII testimony, and donor records converge on the longitudinal pattern. The KP GMB workshop identified a peak financing window of 1979–2009, during which BHUs operated at above 90% capacity. The Sindh workshop located the principal contraction in 1994, when UNHCR pulled back funding after the formal end of the Afghan and Soviet conflict, with partial recovery during COVID‐19 via reinvestment by UNHCR and the International Organisation for Migration. The ICT workshop noted that international funding has fallen sharply since 2000, in line with the broader contraction in humanitarian space. One ICT‐based key informant summarised the magnitude:

There was a time when the annual budget for refugees was 400 million dollars, but now it is less than 4 million dollars over a period of 40 years, and this is only for operational expenses, not activities. The donor fatigue is evident as the focus has shifted to newer crises.

(ICT KII‐06)

Beyond the headline contraction, participants described the diffusion of financial responsibility across administrative tiers as a separate but compounding problem. A KP GMB participant captured the obscuring effect of this diffusion:

Finances were slowly and gradually integrated into many layers of government and bureaucratic matters, which obscured the financial investments towards the real matter of healthcare provision.

(GMB‐KP)

The 2014 redirection of foreign funding from dedicated refugee facilities to general public sector strengthening through RAHA is a documented policy fact [31]. The KP GMB workshop traced the downstream effects: designated refugee health centres lost funds, medicine supply chains collapsed, skilled staff were progressively replaced by unskilled personnel, and door‐to‐door services were withdrawn by 2014. The ICT workshop additionally linked the timing to the diversion of funds towards Syrian, Yemeni, Iraqi, and Burmese refugee responses. UNHCR provides emergency cash assistance of PKR 45,000 per family for acute medical situations, channelled through the CAR. Participants converged on its inadequacy for tertiary‐level care, surgical procedures, and chronic‐disease management, a point made directly by one KP informant:

For medical emergencies, UNHCR provides emergency cash assistance of PKR 45,000 per family, but this amount is not enough to cover actual healthcare expenses.

(KP KII‐15)

The Medical Teaching Institutions (MTI) Act of 2015 made KP teaching hospitals financially autonomous and allowed consultants to treat private patients for a fee while routine services remained subsidized that had previously been available against a nominal Parchi fee of PKR 5 to 10 [32, 33]. Because refugees are not eligible for the SSP, the MTI Act converted de facto inclusion into formal exclusion at the tertiary level, as one informant explained:

Under the Sehat Card scheme, public hospitals can now charge their own fees. For the host community, the Sehat Card covers these costs, but refugees receiving services at tertiary hospitals, specifically teaching hospitals, have to pay for them. This leaves refugees with no choice but to seek required services at private hospitals or explore other means.

(KP KII‐07)

### 3.4 Service Provision

At the primary level, camp‐based BHUs and RHCs are the closest formal point of contact for camp‐based refugees and serve as their first point of entry to the system. Where a BHU is functional, it provides outpatient consultations, EPI vaccination, MNCH services, nutrition support, and TB and malaria screening and treatment. UNHCR‐supported BHUs in KP report utilisation in excess of their original catchment design; a Balochistan BHU originally designed for 10,000 visits per year is reported to be managing up to 60,000. Functionality has declined since 2005 in line with the financing trajectory described above. BHUs are typically the most physically proximate formal facility for camp‐based refugees; however, frequent stock‐outs, irregular opening hours, and progressive de‐skilling of staff have reduced their effective accessibility, so refugees often bypass them in favour of secondary or NGO‐supported facilities even when the latter are further away. UNHCR Pakistan operational data collected during field work in KP between February and March 2024 indicate that 78% of Refugee Villages have a public health facility within 8 km, while 22% lie between 9 and 12 km; transport costs over these distances are unaffordable for daily‐wage workers, and women face additional cultural restrictions on mobility, which raise the cost in maternal and neonatal terms [34]. A Sindh‐based informant captured the resulting unmet need:

There is a huge vacuum to be filled still, as far as the healthcare system and services for these Afghan refugees are concerned; the most prioritised and dire need is the establishment of health care units like Dispensaries, BHUs here in the community for easy access.

(Sindh KII‐02)

The day‐to‐day reality inside camp BHUs reinforces this view, with one camp‐based informant describing the simultaneous collapse of supplies and supporting infrastructure:

We have BHUs, but we don’t have supplies, medicines, contraceptives. We don’t have lab facilities. We also lack transportation facilities for staff.

(KP KII‐13)

A Sindh GMB participant added the corresponding service‐level consequence:

There are no 24/7 facilities in our camp; even basic services like radiology or screening for infectious diseases are unavailable.

(Sindh‐GMB)

Labour rooms staffed by skilled birth attendants were operational in KP refugee camps until 2009, when they were closed. Since then, women in camps have travelled further for skilled delivery; many revert to home deliveries or go without skilled attendance, and the loss of female‐staffed services is a recurrent constraint. A KP informant linked the relocation of facilities directly to falling utilisation:

With health facilities now farther from the camps, they visit these facilities much less frequently, exacerbating the challenges they face.

(KP KII‐04)

A Sindh GMB participant articulated the maternal consequence:

If there is no female doctor, many women simply avoid seeking care, which increases risks during pregnancy.

(Sindh‐GMB)

At the secondary level, District Headquarters (DHQ) and Tehsil Headquarters (THQ) hospitals are the formal referral destination, and refugees are entitled to use them on the same terms as Pakistani citizens upon presentation of a PoR or ACC card. Referrals are inconsistent and largely informal in practice. DHQ Kohat absorbs a substantial proportion of the refugee load, with an estimated 1500 to 2000 of outpatient department volume of 5000 to 6000 being Afghan refugees. The intended pathway was described by one KP informant in straightforward terms:

If a BHU cannot manage a patient’s condition, the recommended action is to refer them to the nearby District Headquarters Hospital (DHQ), conveniently located near the refugee camp. Additionally, there is a Lady Reading Hospital (LRH) in Peshawar, easily accessible to refugees in case of major emergencies.

(KP KII‐04)

Secondary facilities are nonetheless overburdened, with medicine shortages, limited specialist availability, and inconsistent hours. At the tertiary level, urban refugees rely on Lady Reading Hospital, Kuwait Hospital, and Khyber Teaching Hospital in Peshawar; in Sindh, NGO referrals route patients to Jinnah Postgraduate Medical Centre and the Sindh Institute of Urology and Transplantation. Before the MTI Act, refugees accessed these facilities at the nominal Parchi fee; since 2015 they pay fees and remain excluded from the SSP, so tertiary care is increasingly out‐of‐pocket in KP. The boundary at which inclusion ends was explicitly noted:

All refugees have access to all types of health services, same as local citizens in Kohat, there is no discrimination, except for specialised health services.

(KP KII‐07)

Specialised services such as oncology, advanced surgery, psychiatric inpatient care, and fistula repair are practically inaccessible to most camp‐based refugees because of cost, distance, and documentation requirements. The referral system was described across all three provinces as weak and not yet evidence‐based:

We have established a referral system for Afghan refugees to access government healthcare facilities at the secondary and tertiary levels when needed. This system has been recently established and needs to be further strengthened and based on evidence.

(Sindh KII‐01)

Human resources, medicines, and technologies sit within service provision. The Pakistani health workforce is itself constrained, with persistent gaps in physician supply, female cadres, and rural deployment [35]. Refugee‐serving facilities are layered onto this constrained workforce and face two specific issues. First, in camps, an estimated 60%–70% of services are delivered by Lady Health Visitors (LHVs) and nurses. UNFPA‐supported deployment and training of LHVs covers Sexual and Reproductive Health, emergency obstetric care, Long‐Acting Reversible Contraception, Gender‐Based Violence response, and Clinical Management of Rape, and these cadres anchor the referral system to specialist female‐staffed services where they exist. The breadth of this package was described directly by one KP informant:

These LHVs receive training in a comprehensive service package that includes Sexual and Reproductive Health (SRH), emergency obstetric care, Long‐Acting Reversible Contraception (LARC), Gender‐Based Violence (GBV) interventions, and Clinical Management of Rape (CMR). Beyond direct service provision, LHVs are a critical part of the referral system, ensuring that Afghan refugees, especially women and girls, can access specialised care when needed.

(KP KII‐15)

Second, dispensers, vaccinators, and LHVs are retiring without replacement, which progressively de‐skills the BHU workforce; targeted training of midwives from refugee communities, including planned 3‐month maternal and newborn care courses, is among the few sustainable workforce investments now underway. The longer‐term remedy was framed as joint investment in local cadres and infrastructure:

We should focus on training nurses and paramedical staff from the local community, especially in areas where refugees are most vulnerable, such as camps. This will ensure that refugees can easily access healthcare services in the event of an emergency. Moreover, infrastructure development is essential, and proper maintenance and attention are necessary to prevent it from becoming fragile over time.

(KP KII‐12)

Medicines and contraceptives in BHUs serving refugees are unreliably available, with frequent stock‐outs in remote camp settings. Exclusion from the SSP raises particular barriers to high‐cost drugs. One KP‐based key informant described being unable to obtain a PKR 60,000 anti‐cancer drug for a refugee patient that would have been free for a Pakistani citizen:

I have recently been informed by a patient with malignancy that an anti‐cancerous drug is required, costing PKR 60,000, and that is available in HMC hospital free of cost for the host community under Sehat card; however, the refugees are not eligible to get it for free.

(KP KII‐05)

Glucantime for cutaneous leishmaniasis, endemic in some camps, is rarely available through national emergency procurement. Vaccine supply is the system's strongest performer, with high reported coverage of polio and measles among refugees tracking the national decline in wild poliovirus cases between 2014 and 2024.

### 3.5 Monitoring, Evaluation, and Information Systems

The first refugee‐specific data system in Pakistan was a manual UNHCR‐led system introduced in 1996, subsequently computerised, with tools still in operational use. The CAR maintains a Management Information System (MIS) that complements UNHCR's data processes; participants reported that effectiveness has declined as facility functionality has fallen and reporting has lapsed. The District Health Information System (DHIS), the principal national routine data system, does not include a refugee identifier, so refugees who use public sector facilities are aggregated with host‐community users, preventing analysis of refugee‐specific utilisation. The gap was stated directly:

The DHIS is working very effectively and providing detailed reports and data about the local community’s health indicators, but unfortunately, there is no special data column about refugees' health indicators.

(KP KII‐15)

EPI data are the exception. Polio and routine immunisation data are collected daily during campaigns and weekly during routine immunisation, supported by WHO and UNICEF and monitored by the Director of EPI; polio microplanning identifies all under‐five households, including those in refugee villages, even though the system does not maintain PoR‐based identifiers in routine reporting. The strength of this subsystem was emphasised:

The Expanded Programme on Immunisation (EPI) was one of the most successful programs for Afghan refugees, with a benchmark of 98%. The data on polio is live, and our polio‐related staff know about every household that has under‐5 children, and they are provided with an EPI card.

(KP KII‐02)

Outside EPI, the absence of refugee identifiers means that the system is technically intact but functionally inactive at camp level, as one camp‐based informant explained:

We have BHUs, but we don’t have supplies, medicines, contraceptives. We don’t have lab facilities. We also lack transportation facilities for staff. That’s why there is a sense of discouragement among the staff; they are no longer enthusiastic. Due to a lack of these facilities, patients are no longer visiting our facilities, so we are not collecting or reporting any data anymore.

(KP KII‐13)

The sequence was reproduced repeatedly in GMB workshops: input shortages erode staff morale and service readiness; deteriorating services discourage attendance; and with no patient encounters to record, facility‐level reporting to the CAR MIS ceases, whether or not the system is technically operational.

### 3.6 UHC Outputs and Outcomes

#### 3.6.1 Adequacy of Coverage

Adequacy varies by service. Preventive coverage, including EPI, polio, and MNCH outreach, is comparatively high. The longitudinal experience with polio illustrates this:

Since 2014, we haven’t witnessed any polio cases in refugees. Before 2014, it was always our tribal districts or refugees who would have contracted the polio virus, but after 2014, it’s been 10 years we haven’t heard any case in Afghan refugees, which is a good sign. The same is the case with measles.

(KP KII‐06)

Primary curative coverage in camps is constrained by BHU stock‐outs and de‐skilling. Secondary and tertiary coverage is constrained by financial barriers and weak referral pathways. Mental health and specialist coverage is largely absent.

#### 3.6.2 Equity and Inclusion

PoR holders are formally included in primary care and the EPI, TB, and HIV programmes on the same terms as Pakistani citizens. KP‐based informants emphasised the absence of overt discrimination at the point of care:

There is no discrimination in providing services to the Afghan refugees. Naturally, it is not possible in our cultural set‐up that we will deny health services to the Afghan refugees. In fact, we may give them more attention, keeping in mind they are suffering from displacement and war and all those factors.

(KP KII‐09)

ACC holders and undocumented refugees encounter inconsistent eligibility checks at higher levels of care, particularly where digitalised hospital systems trigger CNIC checks. The documentation‐driven nature of this barrier was foregrounded by an ICT GMB participant:

Without valid documentation, even basic health services are hard to access, which adds to the community’s vulnerability.

(ICT‐GMB)

Comparison with the host population is essential: the public system is constrained for everyone, but refugees face additional layers of legal, documentation, and financial exclusion that produce a deeper severity of disadvantage.

#### 3.6.3 Financial Protection

Refugees are excluded from CNIC‐linked schemes, including the SSP, BISP, and out‐of‐pocket payment is the principal financing mechanism for tertiary care. This pattern is consistent with the 2022 HAUS finding that more than 90% of refugees pay out of pocket and nearly 70% are unaware of subsidised public services [29]. UNHCR's PKR 45,000 emergency cash assistance is widely described as a stopgap rather than a financial protection mechanism, a view voiced directly by one KP informant:

For medical emergencies, UNHCR provides emergency cash assistance of PKR 45,000 per family, but this amount is not enough to cover actual healthcare expenses.

(KP KII‐15)

The cancer‐drug case referenced earlier (KP KII‐05) is the clearest illustration of how exclusion translates directly into morbidity and mortality risk.

#### 3.6.4 Quality of Care

Quality is constrained by medicine stock‐outs, the de‐skilling of the camp BHU workforce, the absence of female‐staffed services in some camps, and incomplete referral pathways. The maternal consequence is captured in the Sindh GMB quotation cited above, in which avoidance of care follows from the absence of female providers.

#### 3.6.5 Health Outcomes

Refugee‐disaggregated outcome data are not available in the DHIS, so refugee‐specific outcomes cannot be quantified from national statistics [36]. The convergence of high reported immunisation coverage with the national decline in wild poliovirus cases, from 306 cases in 2014 to a single reported case in 2021, before a modest rise to 14 cases in 2022 in southern KP [37, 38], is the clearest favourable outcome and was directly described by a KP informant (KP KII‐06). Key informants in KP and Sindh linked contaminated water in camps such as Ghamkol to hepatitis C, cirrhosis, and the persistent presence of wild poliovirus in environmental samples, producing a public health concern that is greater than its visible service‐utilisation footprint.

#### 3.6.6 Wider System Effects

Refugee‐adjacent public facilities show a clear strain, articulated directly by one KP‐based informant:

Previously, if we were providing services to 10 individuals from the local community each day, the sudden influx of refugees means we now have to accommodate an additional 5 people, representing a significant 50% increase in demand. This surge in patient numbers puts immense strain on our resources.

(KP KII‐09)

The strain is best understood as additive to a public system already under pressure rather than as a refugee‐specific phenomenon.

### 3.7 Social Cohesion

Social cohesion is the system's most under‐recognised strength. Cultural and linguistic affinity between Afghan refugees and Pakhtun host communities in KP and Balochistan facilitates respectful care; in KP, a 60:40 quota arrangement for refugee and host community access at UNHCR‐supported facilities is widely described as functioning without contestation:

There is no discrimination from the locals and the staff. The quota for accessing healthcare is 60% for the refugees and 40% for the locals has never been questioned.

(KP KII‐01)

Mixed community health committees, jointly convened from refugee and host community members during health awareness programmes, were cited as a vehicle for shared ownership of health initiatives. Intermarriage, language fluency, and absorption into local livelihoods reinforce these arrangements, as one KP informant described:

The majority grew up here; they can speak national languages and travel fluently to different parts of the country. They have married local men and women and are well absorbed in society. However, they still face some discrimination, not only at the health level but in other aspects of life as well.

(KP KII‐01)

Discrimination at the point of care is reported as rare, while social discrimination outside the health system, including the recent rise in repatriation messaging, is described as growing.

## 4 Discussion

This study shows that Pakistan has achieved a pragmatic form of service‐level inclusion without structural integration for Afghan refugees. PoR or ACC holders access public services on terms broadly similar to host communities, but that access rests on administrative goodwill and donor finance rather than on legal entitlement or pooled risk protection. The system is therefore inclusive at the counter and fragmented in its governance and financing spine. This pattern mirrors the global shift since 2016, when the New York Declaration, the Comprehensive Refugee Response Framework, and the Global Compact on Refugees recast refugee health as a shared state responsibility rather than a parallel “care and maintenance” obligation [39]. A critical but underappreciated dimension of Pakistan's refugee health challenge is that many of the barriers Afghan refugees encounter are not refugee‐specific but system‐wide weaknesses, including medicine stockouts, workforce deficits, high out‐of‐pocket spending, and fragmented governance, that affect the host population with comparable severity. Refugee status amplifies these gaps; it does not create them.

The immunisation record, with wild poliovirus cases falling from 306 in 2014 to a single case in 2021 before a modest rise to 14 in 2022 [37, 38] is the clearest proof that aligned financing, surveillance, and frontline integration can deliver equitable preventive care. This is the model the other building blocks should emulate, not the exception that excuses their weakness.

The contrast with curative and protective services is sharp. A foundational cross‐cutting barrier is Pakistan's non‐signatory status to the 1951 Refugee Convention and the absence of domestic refugee legislation, which together create a legal vacuum that radiates outward into every downstream interaction. This mirrors patterns documented globally: across LMIC host countries, insecure or irregular legal status is among the most consistent predictors of exclusion from health insurance, subsidised medicines, and formal referral pathways.

The nine‐domain framework of refugee access barriers captures the compounding: legal, economic, physical, trust‐based, and structural domains all stack under insecure status [40]. In Pakistan, undocumented Afghans additionally face documentation gatekeeping at the point of care, discrimination, and self‐exclusion driven by fear of legal consequences [41]. Documentation‐linked exclusion extends beyond refugees: Pakistani citizens without a CNIC face identical barriers to the SSP, with NADRA's 2022 reporting that rural and urban poor women in KP and Balochistan remain disproportionately unregistered [42]. The gate is the same; it locks both groups out.

The 2015 MTI Act in KP and the SSP rollout illustrate a documented global paradox: when UHC reforms digitise and CNIC‐linked entitlements without an explicit refugee clause, modernisation deepens exclusion. The informal queue that quietly admitted refugees becomes a formal database that pointedly does not [43, 44].

Financial sustainability remains the system's most acute vulnerability. The 2020 UN Office of Internal Oversight Services (OIOS) audit independently confirms the participant accounts of accountability gaps: USD 5.5 million spent over 18 months across 51 parallel facilities, no mainstreaming strategy, and a USD 573,197 Kohat rehabilitation that delivered non‐functional facilities [45]. More recently, the suspension of USAID‐funded programmes in 2025 closed more than 60 health facilities serving 1.2 million Afghan refugees, an illustration of the fragility of any financing model dependent on a single bilateral donor [46]. This sits on top of chronic national underfunding, total health expenditure persistently near 2.9% of GDP [27], which already produces stockouts, staffing gaps, and dysfunctional equipment in DHQ and THQ hospitals nationwide, regardless of refugee presence [28]. The pattern is not unique: Lebanon and Kenya show the same fragmentation between parallel humanitarian and government systems, and Lebanese out‐of‐pocket expenditure for Syrian refugees ranks among the highest globally [47, 48, 49]. Pakistan fits this structural pattern closely.

Medicine stockouts in this study are best read as a symptom of a national supply‐chain disorder: BHU allocations track facility category rather than patient load, more than 55% of public‐sector clinicians and pharmacists report shortages [50], and the Drug Regulatory Authority of Pakistan (DRAP) has formally acknowledged critical shortfalls in secondary care for 2024 [51]. However, the refugee‐specific magnitude cannot be quantified because Pakistan's DHIS carries no refugee identifier, a data invisibility that is itself a finding.

The transition from a well‐resourced UNHCR parallel system to a partially integrated but underfunded one has widened, not closed, these gaps, especially in MNCH care for Afghan women in KP and Balochistan, where funding, coverage, literacy, and provider‐patient cultural distance interact to suppress utilisation [52].

Donor fatigue in protracted displacement is global [53, 54], and the same paradox recurs across LMICs: as host countries digitise insurance, services that were once free at the public counter become conditional on identity documents that refugees lack. WHO confirms that migrants in an irregular status are routinely excluded from national protection schemes [47, 55].

Gender norms compound the financing barrier. Where cultural and religious norms restrict women's consultations with male providers, the shortage of female staff in camp BHUs directly undermines service delivery for SRH, MNCH, and especially for mental health care and suppresses women's care‐seeking for these services [24, 56]. Mental health, therefore, represents an especially significant and largely unaddressed service‐delivery gap. Afghan refugees carry elevated rates of depression, anxiety, and post‐traumatic stress disorder arising from decades of conflict, forced displacement, economic precarity, and repatriation threats. Coercive repatriation tactics, including loudspeaker eviction notices, generate a fear that itself suppresses engagement with health services. Despite this burden, mental health services remain project‐based and episodic, with no systematic integration into routine primary care. The gap mirrors global findings: Syrian refugees in Turkey reported a 14.8% need but only 1.4% service uptake [57], and stigma, language, scheduling, and gatekeeping recur as barriers across settings [58, 59, 60]. In Afghan communities, these structural barriers are further filtered through male authority over women's healthcare decisions. Evidence from Afghanistan shows that when mobile and community‐based outreach teams visit regularly and build trust, women's use of maternal and child health services increases as community confidence grows.

The workforce is the system's paradox. LHVs and community midwives deliver 60%–70% of camp services and carry SRH, emergency obstetric care, and GBV response, yet inconsistent funding, weak supervision, and thin training reportedly contribute to higher neonatal mortality. The same skill‐gap pattern is documented globally as a structural quality limitation in refugee primary care [61, 62]. Comparative evidence is more hopeful: Uganda's deployment of refugee health workers [63] and UNHCR's refugee health volunteer model in Rwanda's Mahama camp both show that refugee‐inclusive workforce strategies strengthen service delivery and economic integration at once [64].

Refugees are also invisible in Pakistan's health data architecture. The DHIS carries no refugee identifier, so utilisation, outcomes, and equity gaps cannot be systematically tracked. WHO treats disaggregated data as the threshold condition for moving from humanitarian to development‐oriented programming [65], and digital platforms such as The Hive [66], machine‐learning applications [67], asset‐based community models [68], and mobile GBV services [69] illustrate what becomes possible once that threshold is crossed.

One Pakistani asset stands out against this picture: comparatively strong social cohesion in KP and Balochistan, where cultural and linguistic proximity makes respectful care easier. Turkey [70], Jordan [71], Lebanon, and Kenya [47, 72]each show how strain and tension can erode that responsiveness, and across these settings, income, education, and legal status remain the strongest predictors of refugee health outcomes. Social cohesion is an enabling condition for reform, not a substitute for it.

Taken together, Pakistan's refugee health barriers are structurally embedded rather than incidental, and the global evidence converges on a single point: neither a purely humanitarian nor a purely state‐led approach delivers sustainable equity. The route forward is genuine integration supported by dedicated and diversified financing, including the Refugee Health Financing Model [19], blended finance, and Islamic instruments such as the Refugee Empowerment Sukuk [73], legal inclusion, and a data architecture that makes refugees visible [44]. Without consolidation, current gains will erode; with it, pragmatic inclusion can mature into durable, system‐wide integration that benefits refugees and hosts together. These conclusions translate into several concrete implications for policy.

### 4.1 Implications for Policy

Six concrete actions emerge from the findings and chart a path to more inclusive, sustainable refugee health. Refugee health should be institutionalised through a federally endorsed SOP that defines entitlements, eligibility, and referral pathways within a single, binding framework. Financial protection must be expanded by including PoR and ACC holders in Sehat Sahulat or creating a dedicated, subsidised refugee health financing window backed by pooled and Islamic financing. Government BHUs in refugee‐hosting districts should be upgraded with more female providers, stronger diagnostics, reliable essential‐medicines supply matched to patient load, and effective referral links. A unified governance platform is needed to convene federal and provincial health departments, UNHCR, CAR, donors, and NGOs in one coordination body that consolidates approvals and keeps health central to migration and repatriation decisions. Frontline workers require targeted training, supportive supervision, predictable incentives, and defined roles for trained Afghan refugees as community health workers. Finally, refugees should be made visible in routine data by adding a refugee identifier to DHIS and harmonising UNHCR‐government information systems so utilisation, outcomes, and equity gaps can be tracked and addressed.

## 5 Conclusion

Pakistan has delivered service‐level inclusion for Afghan refugees without the structural integration that would make it durable. The immunisation record shows the model can work; governance fragmentation, donor‐dependent financing, and exclusion from national social protection show how easily those gains can be undone. Because the binding constraints are shared with the host system, embedding refugee inclusion in national health planning, financing, and information systems will strengthen care for both populations simultaneously.

## 6 Limitations

This study has five main limitations. Its qualitative, three‐site design is well‐suited to exploring health system dynamics but does not permit statistical generalisation. Purposive sampling and a 40‐year retrospective GMB scope introduce recall and social‐desirability bias, and unregistered refugees who are most exposed are likely underrepresented because participation carries perceived risk. Evidence on financing relies on triangulating participant accounts with the 2020 OIOS audit and public donor reports, as disaggregated per capita expenditure and full donor ledgers are not publicly available. The DHIS does not disaggregate by refugee status, preventing quantitative corroboration of access and outcome patterns. Finally, fieldwork (April‐June 2024) preceded the 2025 USAID suspension, so the analysis reflects the conditions that produced the current crisis rather than the crisis itself.

## Funding

Funding for this research was granted by the United Nations' High Commissioner for Refugees (UNHCR), under award 2022_RFP_002. This work was led by Queen Margaret University, Edinburgh, under grant number P2079QMU.

## Conflicts of Interest

The authors declare no conflicts of interest.

## Data Availability

The data that support the findings of this study are available on request from the corresponding author. The data are not publicly available due to privacy or ethical restrictions.
